# Laser Surface Treatment of Polymethacrylate Materials for Biocompatibility Improvement

**DOI:** 10.3390/polym18121425

**Published:** 2026-06-07

**Authors:** Ann V. Gritsaeva, Ivan A. Popov, Dmitriy A. Serov, Ivan A. Novikov, Anastasiia V. Shabalina, Dmitriy E. Burmistrov, Alevtina G. Nesterova, Sergey V. Gudkov, Valery A. Kozlov

**Affiliations:** 1 Prokhorov General Physics Institute of the Russian Academy of Sciences, Vavilov Str. 38, 119991 Moscow, Russia; anngritsaeva@mail.ru (A.V.G.); popovia2003@yandex.ru (I.A.P.); dmitriy_serov_91@mail.ru (D.A.S.); i.novikov@niigb.ru (I.A.N.); shabalinaav-1985@yandex.ru (A.V.S.); dmitriiburmistroff@gmail.com (D.E.B.); s_makariy@rambler.ru (S.V.G.); 2 Krasnov Research Institute of Eye Diseases, 119021 Moscow, Russia; 3 Department of Chemical Technology of Polymeric Composite Paints and Coatings, Mendeleev University of Chemical Technology, Miusskaya Sq. 9, 125047 Moscow, Russia; nesterova.a.g@muctr.ru; 4 Department of Fundamental Sciences, Bauman Moscow State Technical University, 5 2nd Baumanskaya St., 105005 Moscow, Russia

**Keywords:** polymethacrylates, laser surface treatment, laser-induced patterns, biocompatibility, cytotoxicity, antibacterial

## Abstract

Methacrylate-based materials, widely used in dentistry, must possess high biocompatibility with oral cells and tissues. Currently, to improve the integration of orthodontic devices with the biological structures, laser-assisted polymer modification is actively employed. Importantly, functionalization is required only for the surface of the material that directly interacts with the oral tissues. This study presents approaches for laser modification of polymethacrylate materials and evaluates their influence on the proliferative activity of human spleen fibroblasts. Using laser radiation, two geometric patterns were obtained on the polymer surfaces. Cell morphology and proliferation on the experimental samples were assessed using scanning electron microscopy. It was found that the polymer with a groove-textured surface (pattern 1) promoted enhanced cell adhesion and reduced material toxicity. Additionally, the antibacterial properties of the polymers were evaluated. The sample with sparsely distributed surface craters (pattern 2) demonstrated an antifouling effect against *Escherichia coli*.

## 1. Introduction

Polymethacrylate (PMA) products, obtained from methacrylate resin, are widely used in modern dentistry, particularly in the fabrication of aligners, dentures, temporary crowns, and various orthodontic structures [1,2]. These materials attract significant interest due to their acceptable cytocompatibility, durability, low cost, and ease of use. Unfortunately, during the manufacture of polymethacrylate products, incomplete polymerization is possible, with the retention of methacrylate monomer and photoinitiator residues in the bulk and on the surface of the finished product [3,4]. In such cases of insufficient polymerization, the polymers may exhibit cytotoxicity due to the release of underpolymerized monomers [5,6,7,8,9]. When such products come into contact with the oral mucosa, there is a risk of toxic effects on surrounding cells and tissues of the body [10,11]. It is important to note that biocompatibility should primarily be possessed by the polymer surface which directly comes into contact with body tissues [12]. Polymer surfaces, especially structured ones, can serve as a favorable substrate for bacterial growth or, conversely, exhibit antibacterial activity [13]. The surface properties of materials also determine not only their bioactivity but also the antimicrobial properties of dental materials [14,15,16]. Currently, the medical industry is actively engaged in research and development aimed at modifying orthodontic devices with the aim of, on the one hand, improving the adhesion of human cells to the polymer surface and, on the other hand, ensuring high antimicrobial properties. The surface modification of polymeric products can be achieved in various ways: chemical modification, coating materials with synthetic and natural polymers, physical treatment, and hybrid approaches to enhancing the bioactivity of the products. Chemical methods aim to alter the chemical composition of the surface or to create active groups on the surface that specifically bind to biological entities and promote cell adhesion, osseointegration and the manifestation of antimicrobial properties [17,18,19]. Thus, to reduce the adhesion of *Candida albicans* to poly (methyl methacrylate) (PMMA) surfaces, the authors employed two methods of modifying the dental resin: altering the surface charge through the copolymerisation of methyl methacrylate with methacrylic acid, and applying a self-crosslinking polymer to the PMMA surface [20]. In [21], the researchers modified the surface of a composite dental resin using the photoreactive polymer 2-methacryloyloxyethyl phosphorylcholine (MPC), thereby preventing the adsorption of mucin and, consequently, the formation of a bacterial biofilm. Surface modification of materials using bioactive and functional coatings [22], including protein and collagen compounds [23], carbon-based nanomaterials [24,25], and calcium phosphate components [26], stimulates cellular activity and accelerates bone tissue integration. A coating containing TiO_2_ nanoparticles, applied to PMMA samples using a spraying method, demonstrated high biocompatibility and proved to be non-toxic to oral tissues [27]. Physical methods include techniques aimed at changing the surface microtopography through mechanical action [28,29,30,31]. For example, in the study [32], researchers presented a method of Ar/O_2_ plasma treatment of the surface of dental materials based on acrylic resins, which improves the surface hydrophilicity by cleaving the original functional groups and forming new hydrophilic groups on the surface of the materials, thereby significantly reducing the adhesion of *Candida albicans*.

Currently, the modification of surface structures using photonic technologies is a sought-after approach in materials development aimed at enhancing biocompatibility. Laser processing is considered an environmentally friendly and precise method that does not require the use of chemical dopants. This method allows for the creation of precisely controlled micro- and nanotopographies: grooves, pits, craters, and various periodic surface structures [33]. Laser ablation forms a deep, regular relief, which dictates the directional orientation of cells along the created structures and promotes the activation of proliferative processes, ensuring effective adhesion of biological objects to the surface [34,35]. The choice of laser pulse duration plays a decisive role in the ablation mechanism of the polymer matrix [36]. A short pulse duration (e.g., picoseconds, femtoseconds) enables the generation of more precise nano- and microreliefs without significant thermal damage around the ablated area [37]. Short pulses are also often used to modify transparent polymer materials due to the occurrence of multiphoton absorption and the incubation effect [37,38]. In turn, picosecond pulse durations occupy an intermediate position between nanosecond and femtosecond ones, which determines their unique advantages: significantly less thermal damage compared to nanosecond ones and at a significantly lower cost compared to femtosecond systems. In the work [39], the surface of a medical implant was modified using nanosecond and sub-picosecond laser processing modes. The authors found that nanosecond ablation resulted in the formation of crown-like projecting rims around the holes, while switching to sub-picosecond pulses eliminated this undesirable effect. The transition from nanoseconds to shorter pulse durations significantly reduces thermal side effects (e.g., the formation of melted beads/rims), ensuring cleaner microprocessing. On the other hand, femtosecond systems are significantly more expensive than picosecond ones in both acquisition and operating costs [40]. However, picosecond laser systems provide submicron resolution and high energy efficiency for microtexturing [41,42]. Thus, laser ablation with picosecond lasers has become an efficient and cost-effective way to produce high-precision structures on polymer surfaces with a reduced heat-affected zone and less contamination around the ablation zone. For example, researchers used picosecond laser ablation (a picosecond pulse Nd:YVO_4_ laser, the pulse duration τ = 10 ps) to apply microstructures (craters and grooves) to the surface of polyhydroxyalkanoates (PHAs) in order to improve the biocompatibility of the material for its use in tissue engineering, as well as to reduce bacterial proliferation [42]. However, this work does not include a biological experimental part, which can be considered a shortcoming of the study. In the study [43], using picosecond laser processing (a picosecond laser system NL-LG-4310, the wavelength λ = 1064 nm, the pulse duration τ = 15 ps), the researchers controlled the hydrophilic–hydrophobic properties (contact angles 8–90°) of the polycarbonate (PC) surface and assessed cell adhesion: PC with moderate hydrophilicity (32–48°) demonstrated significant cell attachment compared to the original sample surface (83°, low hydrophilicity), superhydrophilic PC (8°) showed anti-adhesive properties. However, the authors, inspired by the natural microstructure of animal surfaces, limited themselves to a pattern of one geometry: the laser-induced periodic pillar-like structures. From a scientific perspective, it would be interesting to examine the hydrophilic properties of PC on patterns of other geometries, as well as to study in more detail the adhesion of biological objects to the material’s surface (e.g., using scanning electron microscopy (SEM) and confocal microscopy). On the other hand, using a shorter duration may result in a less pronounced relief compared to picosecond laser processing. Thus, in [44], nanosecond laser treatment (a pulsed Nd:YAG laser, the pulse duration τ = 10 ns) of the polycarbonate (PC) surface improves the adhesion of the mouse fibroblast cells. Improved hydrophilicity and significant cell attachment are characteristic of samples with reliefs 1 and 2, obtained using a wavelength of λ = 1064 nm, beam diameter d = 0.8 mm, 5 and 1 pulses per point, respectively, and having a valley-type morphology. Morphological observations of the surface are limited only to SEM images, and they reveal a rather chaotic relief, without a pronounced structure over the entire surface area of the material. Also, since the work is aimed at material biocompatibility in tissue engineering, it makes sense to test human cell culture on experimental samples.

It is worth noting that there are few studies in the literature on the use of picosecond ablation for surface patterning of PMMA/PMA-like polymers, which are frequently used in the dental field, or these studies are limited by the lack of necessary biological experiments and/or microbiological studies, investigating only one of the numerous parameters (one and/or several geometric patterns, hydrophobic–hydrophilic characteristics) [43,45]. Currently, the overwhelming majority of studies, including cellular experiments on modified PMMA surfaces, are focused on the use of lasers with shorter pulse durations or the use of a continuous mode of operation. In a study [46], ordered micrometric parallel grooves were created on the surface of PMMA using laser radiation (InGaAIP diode laser, the wavelength λ = 660 nm), resulting in improved adhesion and faster cell growth. However, characteristic overgrowth along the created structures on patterns of this type is not observed in the presented images. In another study [47], CO_2_ laser texturing of polymers prevents microbes from attaching to the surface of materials and, as a result, minimizes the subsequent formation of biofilm. Thus, the creation of rectangular patterns of different sizes (~10 s of μm) on the surface of PMMA led to a 53.7% reduction in the density of *Escherichia coli* (*E. coli*) bacteria compared to the non-textured sample.

These methods of modifying material surfaces enhance biocompatibility with recipient tissues by altering the wettability, surface charge, and hydrophobic and hydrophilic properties of the medical device, as well as by creating roughness, micro- and nanostructures on the material surfaces. This leads to changes in the degree of adsorption of cells and proteins involved in the biological response to the implanted specimens. Surface topography modification of dental structures represents a promising approach to enhancing the biocompatibility of materials and significantly reducing the pathogenic activity of bacteria.

Our study aims to assess the influence of laser-induced microrelief of PMA surfaces on their biocompatibility with human cells, including cell adhesion, as well as the potential antibacterial effect upon contact with material surfaces. This research includes a comprehensive study covering picosecond laser texturing of PMA polymers with the application of two types of microrelief, conducting biological experiments assessing the effect of polymers in the presence of human cells, as well as during their contact interaction, and investigating the potential antibacterial effect of the materials. Our use of patterns of two geometries, namely grooves and spots with rare craters on the surface, will help evaluate the biocompatibility of the material in contact with human cells, as well as examine the nature of human cell adhesion along the created structures. We can study whether grooves (pattern 1) affect the specified directional growth of fibroblasts along these structures [34], and how the rare craters on the material surface during spot formation (pattern 2) facilitate cell spreading in the absence of significant changes in surface morphology. The results of the study show that material surface microtexturing using laser radiation has a beneficial effect on the growth of cell cultures and significantly improves the adhesion of biological objects to PMA substrates with a modified surface (pattern 1). A supplementary investigation involved the evaluation of the antibacterial properties of polymethacrylates with a specified surface texture and a comparison of these with a control polymer.

## 2. Materials and Methods

### 2.1. Sample Preparation

**Figure 1:**
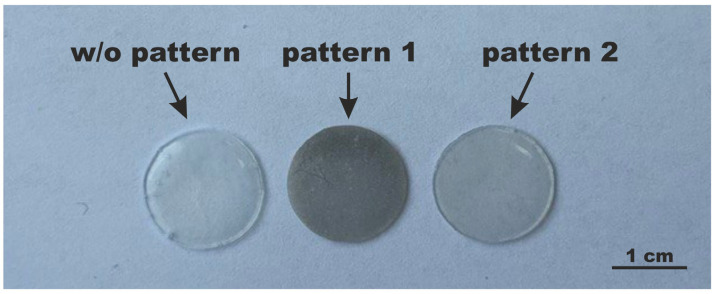
Polymethacrylate samples without a pattern and with patterns 1 and 2, obtained using laser radiation.

The experimental samples were made from methacrylate dental resin Dental Splint (Harz Labs, Mytishchi, Russia) using the casting method, followed by polymerisation and post-processing. The methacrylate resin was pipetted into 15.8 mm diameter silicone molds. A 405 nm laser module was used for cross-linking. The post-treatment consisted of the following steps: rinsing the polymers in isopropyl alcohol 99.9% (Diam, Moscow, Russia) in an ultrasonic bath (Super Accomplish Health Technical Co., Ltd., Fuzhou, China) for 6 min, curing in a drying oven (Xieli International Trading Co., Ltd, Wanchai, China) at 80 °C for 15 min, subsequent exposure in a Magnum Dental Flash PRO ultraviolet polymerization chamber (Magnum 3D, Moscow, Russia) and final processing in a drying oven at 80 °C for 30 min. A PL PDP-3114 SH picosecond laser (Synchrotech Ltd., Moscow, Russia) was used to change the surface relief of the samples. The following radiation parameters were used: wavelength λ = 1064 nm, pulse duration τ = 30 ps, repetition rate ν = 1 kHz, and pulse energy *E* = 2.5 mJ. The microrelief on the surface was created by moving the focused beam along the target surface using an LScanH galvanometer scanner (Ateco-TM, Moscow, Russia) and an F-Theta lens with a focal length of 90 mm. The laser spot at the focus was 30 µm. The beam waist diameter was measured by varying the focal length and determining the ablation crater diameter. The energy density (fluence) was 354 J/cm^2^. The corresponding peak power density was approximately 1.18 × 10^13^ W/cm^2^. The beam movement speed using the scanning system was 60 mm/s. The typical time required to form the microrelief was 2 and 1 min for each pattern, respectively. Thus, using photonics technologies, two types of geometric patterns were created on the polymer surfaces: pattern 1—grooves 75 μm wide, spaced 45 μm apart; pattern 2—spots with a diameter of 30 µm spaced 80 µm apart along the material (Figure 1). Samples with the resulting textures were treated twice in isopropyl alcohol in an ultrasonic bath (see above) for 5 min to ensure sterility and for further use in biological experiments.

The accuracy of the microrelief was assessed using an upright optical microscope MX6R-RT (AMADA, Shanghai, China) and a scanning electron microscope SM-32 (Melytec, Moscow, Russia).

### 2.2. Measuring the Contact Angle

Measurements were performed using the sessile drop method on an analyzer equipped with a stage with precise movement, a Wanptek DPS605U power supply, and an LED-illuminator with diffusing optics. The experiment was conducted at a temperature of 24 ± 2 °C and a relative humidity of 50 ± 10%. The purified materials were placed on the stage, and a 5 μL drop of distilled water was then applied to the surface of the samples. Droplet images were recorded using an SDU3-S264 CMOS camera (SpetsTeleTekhnika, Moscow, Russia). Ten independent measurements were taken for each experimental material. Image analysis and contact angle (θc) calculation were performed using ImageJ 1.54p software (Fiji) (National Institutes of Health, University of Wisconsin, Bethesda, WI, USA).

### 2.3. Biological Tests

Biological experiments included cytotoxicity assessment using MTT assay and fluorescence microscopy. Human spleen fibroblast cell culture (HSF, #5530, ScienCell, Carlsbad, CA, USA) was used to investigate biocompatibility. The cells were cultured and passaged in accordance with standard protocols. DMEM/F12 supplemented with 10% FBS, 25 units/mL penicillin, 25 μg/mL streptomycin and 2 mM L-glutamine (PanEco, Moscow, Russia) was used as the culture medium. For further use in experiments, the cells were detached from the surface of the culture flask using a Trypsin-Versene solution. Then the cell concentration was determined by counting in a hemocytometer (Neubauer chamber). HSF cultured for 5–10 passages were used for the experiments.

To assess the mitochondrial activity of viable cells cultured in the presence of modified materials, a standard MTT colorimetric assay was performed. Sterile polymethacrylate samples were pre-loaded into a culture plate. To investigate the effect of the materials on the metabolic activity of biological organisms, cells were seeded into a culture plate at a density of 100,000 cells per well in 1 mL of culture medium. The experimental samples with cells were placed in a S-Bt Smart Biotherm CO_2_ incubator (Biosan, Riga, Latvia) for 3 days. After 72 h of incubation, 300 μL of a working solution of MTT [3-(4,5-dimethylthiazol-2-yl)-2,5-diphenyl tetrazolium bromide] (Diam, Moscow, Russia) at a concentration of 0.5 mg/mL was added to each well with pre-selected culture medium and materials. The HSF cell line was then incubated with the MTT solution for 4 h to allow the dye to be reduced to insoluble formazan. The amount of formazan formed is proportional to the number of viable cells. After incubation, dimethyl sulfoxide (DMSO) (LenReactiv, Moscow, Russia) was added in a 1:3 ratio (900 µL) to dissolve the formazan crystals. To ensure thorough mixing, the plate was left on a shaker for 5 min at 300 rpm and additionally resuspended using a pipette. The optical density of the samples was measured at 555 nm using a Feyond-A300/A400 microplate reader (Hangzhou Allsheng Instruments Co., Hangzhou, China).

Cell viability was assessed using fluorescence microscopy. A cell suspension containing 100,000 cells was applied in a drop of culture medium (100 µL) onto the surface of sterile 24 mm diameter coverslips (Minimed, Moscow, Russia), one per well of a 6-well culture plate, and incubated for 30 min to allow cell adhesion. Then, 2 mL of culture medium was added to each well, and the experimental polymer samples were immersed nearby. To investigate the effect of the materials on the cell line, these samples with cells were incubated for 3 days in a CO_2_ incubator. Viability analysis was performed using fluorescent probes: Hoechst 33342 and Propidium Iodide (PI) (all Lumiprobe, Hunt Valley, MD, USA). Before cell staining, the polymers were removed from the culture plate. Cells were stained with 2 μg/mL Hoechst 33342 for 30 min at 37 °C in an incubator. The cell slides were placed in a special coverslip chamber RC-40LP (Warner Instruments, Hamden, CT, USA), washed twice with phosphate-buffered saline (PBS), and stained with 2 μM PI for 1 min. The chamber and slide were placed in a special stand and analyzed on a DMI4000 B fluorescence microscope (Leica, Wetzlar, Germany) equipped with an SDU-285 digital camera (SpetsTeleTekhnika, Moscow, Russia). WinFluorXE software v 3.8.7 8-12-16 (J. Dempster, Strathclyde Electrophysiology Software, University of Strathclyde, UK) was used for data collection. Hoechst 33342 stained live and dead cells, while PI stained only dead cells. Images obtained during the experiments were processed using ImageJ 1.54p software (Fiji) (National Institutes of Health, University of Wisconsin, Bethesda, WI, USA). Binary image masks were generated, specific cellular regions were isolated by filtering the images by brightness, and cell viability was calculated. At least five samples were analyzed for each experimental variant. 250–500 cells were analyzed in each sample. The method is described in more detail in [48]. Additionally, viability was assessed using fluorescence microscopy on the fibroblast-like cell line NCU-F8 (collection of All-Russian Research Institute of Medicinal and Aromatic Plants VILAR, Pushchino, Moscow, Russia), isolated from human oral mucosal epithelium under similar conditions in order to compare survival rates on two cell cultures (HSF and NCU-F8).

### 2.4. Scanning Electron Microscopy

Cell proliferation was assessed using scanning electron microscopy (SEM) on a microscope SM-32 (Melytec, Russia). The surface characteristics of the cell-free materials were evaluated using both secondary and backscattered electron detectors. Cultivation of biological specimens on the experimental samples lasted 1 day (24 h) and 4 days (96 h). Fixation and staining of HSF were carried out according to the BioREE™ method (Glaukon LLC, Moscow, Russia) using the BioREE-A kit, consisting of three components [49]. A contrasting solution based on neodymium chloride (NdCl_3_) was used in this set. Each component of the kit was added sequentially to the well plates containing the experimental samples with cell cultures and incubated in accordance with the instructions. The surface of experimental samples with biological structures contrasted with lanthanides was studied in the backscattered electron mode, which made it possible to visualize the internal structure of the cells and evaluate their distribution on the substrate.

### 2.5. Bacterial Growth and Biofilm Formation Assay

Several standard methods were used to investigate the antibacterial properties of the materials obtained: the disc diffusion method and a biofilm formation test (SEM). Prior to the experiments, the samples were treated with 70% ethanol, then with a phosphate–saline buffer solution, and sterilised using ultraviolet radiation for 30 min.

#### 2.5.1. Disk Diffusion Method

The antibacterial activity was assessed using the standard agar diffusion method. For the experiments, a bacterial culture of *Escherichia coli* strain BL21 was used. The bacteria were cultured in standard Lysogeny broth (LB) according to Miller (Diam, Moscow, Russia) at 37 °C with constant agitation at 230 rpm in an ES-20 orbital shaker-incubator (Biosan, Riga, Latvia). Immediately prior to the experiment, the culture was transferred to fresh nutrient medium and incubated in a shaker at 37 °C and a speed of 230 rpm for 24 h. In parallel, freshly prepared and autoclaved (120 °C, 30 min) Mueller–Hinton agar (MHA) (Diam, Moscow, Russia) was poured into sterile Petri dishes and left to solidify completely under sterile conditions. A bacterial suspension (10^6^ CFU/mL) was then spread in several directions across the entire surface of the agar-containing dish using a Drigalski spatula. The material without laser treatment and samples with patterns 1 and 2 were placed with the modified surface facing the Petri dish containing the bacterial culture. After 48 h of incubation at 37 °C, the antibacterial effect was assessed based on the presence of growth inhibition zones around the polymethacrylates tested. The experiments were carried out in duplicate.

#### 2.5.2. Assessment of Biofilm Formation (SEM)

A biofilm formation test was conducted for *E. coli* in six-well plates. The test samples were placed directly into the wells of the culture plate. Two millilitres of LB broth containing 10^5^ CFU of bacterial cells were added to each well. The six-well plates were incubated at a constant temperature of 37 °C for 24 h and 96 h to assess the degree of biofilm growth. The bacterial culture samples were then removed from the plates and fixed for SEM. The biological specimens were subjected to a two-step staining procedure using the BioREE-B kit, which included additional lead salt counterstaining. A quantitative analysis of the obtained SEM images was carried out with ImageJ 1.54p software (Fiji) (National Institutes of Health, University of Wisconsin, Bethesda, WI, USA).

### 2.6. Statistical Analysis

Experimental data were processed using MS Office Excel v. 14.0.6023.1000 and OriginPro 2021 v. 9.8.0.200 (OriginLab Corporation, Northampton, MA, USA). Data were presented as mean values ± standard error (SE). Statistical hypotheses were tested using Kruskal–Wallis ANOVA with a post hoc Dunn’s test. Differences were considered statistically significant at a significance level of *p* < 0.05. Exact sample sizes are provided in the figure legends.

## 3. Results and Discussion

### 3.1. Microrelief Characteristics

Experimental round-shaped samples made of methacrylate resin are shown in Figure 1.

Two types of patterns on the PMA surface were obtained by varying parameters such as pulse energy and pulse numbers (number of laser pulses per local region of polymer surface during scanning). In our study, measurements were carried out at a constant pulse frequency ν = 1 kHz and scanning speed V = 60 mm/s.

**Figure 2:**
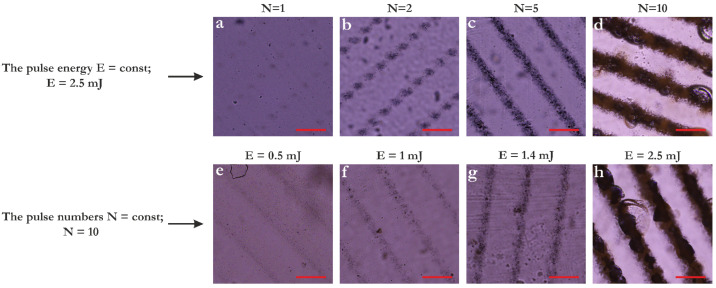
The effect of changing ablation parameters on the surface geometry of the PMA. Laser ablation was performed under two conditions: fixed pulse energy *E* and variable number of pulses *N*: *N* = 1 (**a**), *N* = 2 (**b**), *N* = 5 (**c**), *N* = 10 (**d**); and fixed number of pulses *N* and variable pulse energy *E*: *E* = 0.5 mJ (**e**), *E* = 1 mJ (**f**), *E* = 1.4 mJ (**g**), *E* = 2.5 mJ (**h**). Images obtained using optical microscope MX6R-RT (AMADA, Shanghai, China). The scale bar: 100 μm.

The scanning speed was chosen such that adjacent laser spots did not overlap, and it did not change during the experiments. Upon single-pulse exposure with an energy of 2.5 mJ, slight changes in PMA structure were detected (Figure 2a). At *N* = 2 pulses, an irreversible surface modification of the material begins to be observed, accompanied by the formation of spots (Figure 2b). As the number of pulses increases, we observe a gradual merging of the spots, leading to the formation of continuous grooves. However, after 10 pulses, deep grooves with clearly defined geometry are formed (Figure 2d). On the other hand, experiments with a fixed number of pulses (*N* = 10) and varying pulse energy did not yield fundamentally different geometries (Figure 2e–h). With decreasing energy, grooves without noticeable depth are observed at 0.5 mJ (Figure 2e). Therefore, in our study, the number of pulses *N* was chosen as the main variable at a fixed pulse energy of *E* = 2.5 mJ. This allowed us to obtain two fundamentally different geometries: grooves (pattern 1) and spots with occasional craters extending to the surface (pattern 2).

**Figure 3:**
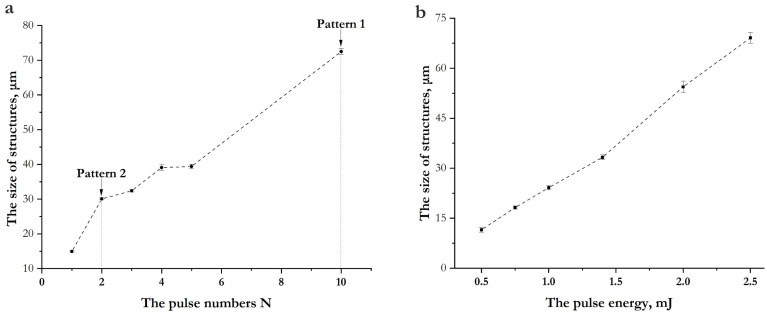
Dependence of the size of structures obtained during ablation on the number of pulses (**a**) and the pulse energy (**b**). Data are presented as mean values ± SE (*n* = 10).

To quantitatively represent the obtained data, we plotted the size of the resulting structures (diameter for spots and width for grooves) as a function of the pulse numbers (Figure 3a). The values corresponding to the selected geometries 1 (grooves) and 2 (spots) are indicated in the graph.

Additionally, a graph was provided showing the dependence of the size of structures on the pulse energy (Figure 3b). The ablation threshold values were calculated using the formula $F_{th}=\frac{E}{\pi \cdot r_{0}^{2}}$, where *E*—the pulse energy in J, $r_{0}$—the beam radius in cm. The absence of ablation at *N* = 1 allows us to identify the lower limit: $F_{th}$ > 71 J/cm^2^. The first laser pulses do not lead to instantaneous ablation of the material, but contribute to the accumulation of structural changes in the material [50]. As the number of pulses increases, the ablation threshold of the material decreases. Varying the energy with a fixed number of pulses does not result in the emergence of new surface geometry.

**Figure 4:**
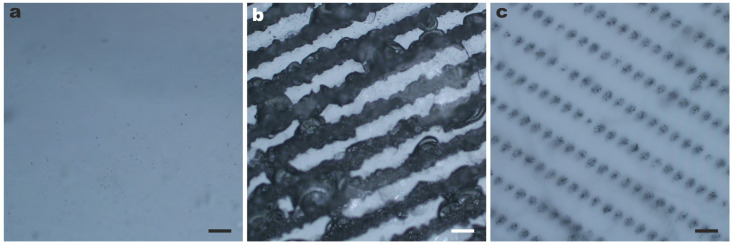
Images of polymethacrylate surfaces obtained using an optical microscope: (**a**) sample without a pattern; (**b**) sample with pattern 1; (**c**) sample with pattern 2. Scale bar: 100 μm.

**Figure 5:**
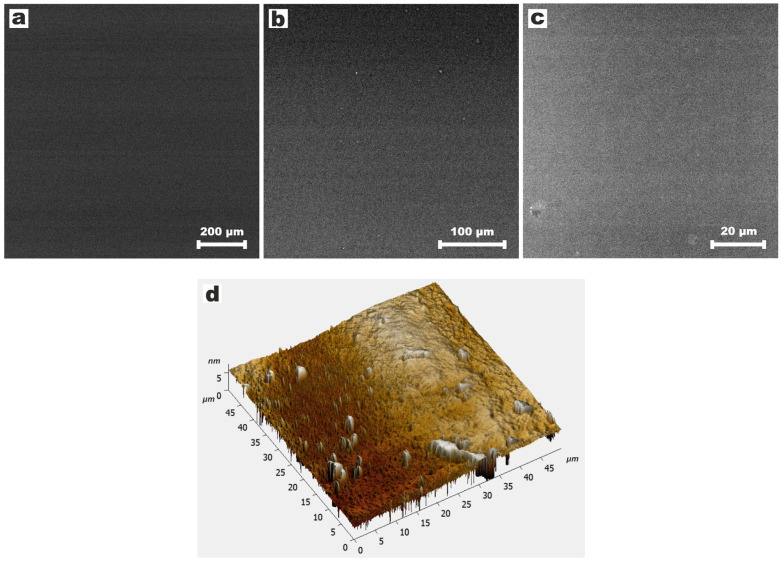
SEM images of the surface of untreated polymethacrylate at different magnifications; scale bars: (**a**) 200 µm, (**b**) 100 µm, (**c**) 20 µm; AFM image of 50 × 50 μm surface area on untreated polymethacrylate (**d**).

The original and modified surfaces of the polyacrylate materials were studied using optical microscopy and scanning electron microscopy with secondary (SE) and backscattered (BSE) electron detectors. Optical microscope images of the samples are shown in Figure 4. Figure 4a and Figure 5 show that the untreated surface of the material is smooth, even, and without significant roughness. Additionally, the surface of the untreated PMA was examined using atomic force microscopy (AFM). A three-dimensional reconstruction of the polymer surface is shown in Figure 5d. The PMA without a laser-induced pattern had a relatively smooth surface without significant defects, and the profile roughness along the Y axis did not exceed 5 nm.

**Figure 6:**
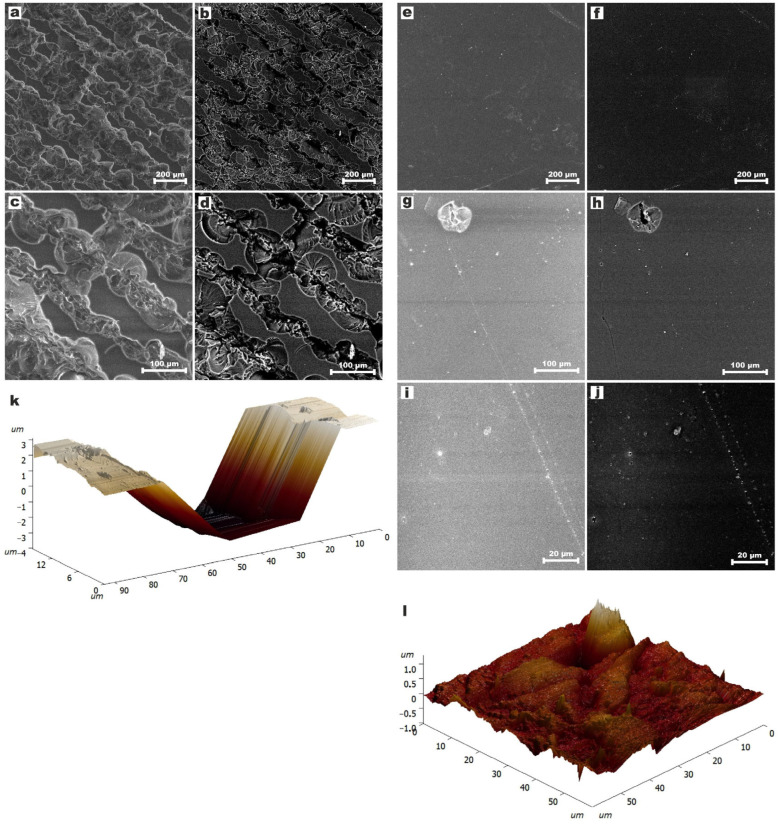
SEM images of the surfaces of polymethacrylate samples with patterns 1 and 2, obtained using laser technology, at different magnifications. (**a**,**c**) Images obtained by secondary electron detection (morphological contrast) for the surface of the material with pattern 1 (scale bars 200 µm and 100 µm respectively); (**b**,**d**) images obtained using backscattered electron detection (z-contrast) for the surface of the material with pattern 1 (scale bars 200 µm and 100 µm, respectively); (**e**,**g**,**i**) images obtained by detecting secondary electrons (morphological contrast) for the surface of the material with pattern 2 (scale bars 200 μm, 100 μm and 20 μm respectively); (**f**,**h**,**j**) images obtained by backscattered electron detection (z-contrast) for the surface of the material with pattern 2 (scale bars 200 μm, 100 μm and 20 μm, respectively). AFM images of 90 × 16 μm surface area on PMA with pattern 1 (**k**) and 60 × 60 μm surface area on PMA with pattern 2 (**l**).

Meanwhile, the polymer with pattern 1 is characterised by shallow, elongated grooves distributed periodically across the entire surface (Figure 4b and Figure 6a–d). AFM imaging of PMA with pattern 1 was performed (Figure 6k). Although the groove depth exceeded the AFM vertical range, the images clearly show that the ablation depth is at least 7 μm. AFM also confirms that the groove width is on average 75 µm. Morphological observations of the surface of the sample with pattern 2 showed that rounded structures form in the near-surface layer of the material, with only minor crater formation directly on the surface itself (Figure 4c and Figure 6e–j). In turn, AFM images demonstrate a crater depth of 1 μm in areas of the PMA surface measuring 30 μm (Figure 6l).

To study the effect of surface modification on the hydrophilic–hydrophobic characteristics of the materials, the wettability of the surfaces was evaluated by measuring the wetting edge angle.

**Figure 7:**
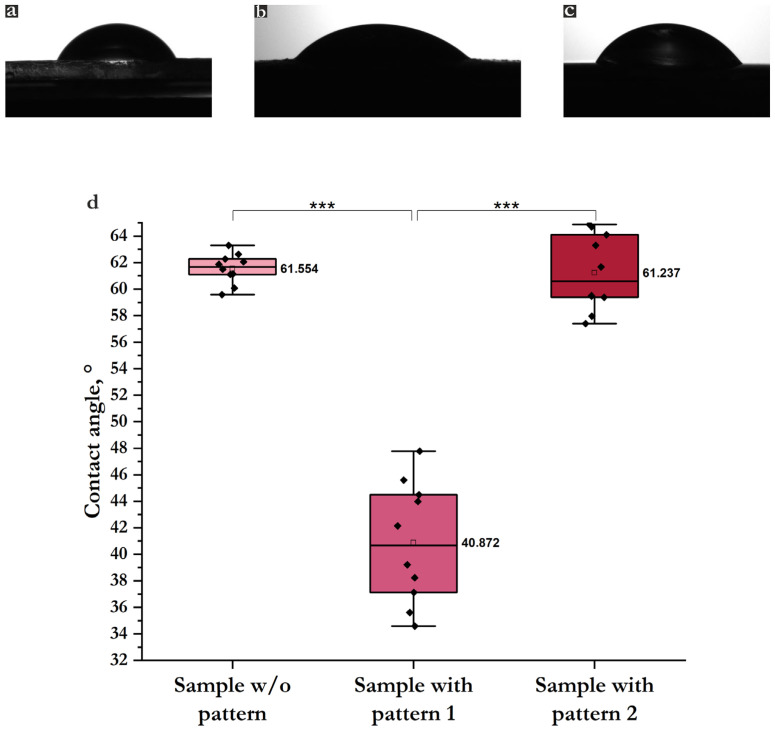
Results of determining the contact angle for the surfaces of untreated PMA and PMA with patterns 1 and 2. Representative images of water droplets on the surface of PMA without a pattern (**a**), PMA with pattern 1 (**b**) and PMA with pattern 2 (**c**) were obtained; the average contact angle was calculated for PMA without a pattern, PMA with pattern 1 and PMA with pattern 2, respectively (**d**). The data are presented as a box plot, where the boundaries of the box are the 25th and 75th percentiles, the line inside the box represents the median, the transparent square (□) represents the average value with a number next to it, and the whiskers extend to the highest and lowest values within 1.5 × IQR (interquartile ranges) of the corresponding quartiles. The sample sizes *n* = 10. The shown *p*-values were calculated based on Kruskal−Wallis ANOVA test results: ***—*p* < 0.001.

The results of the hydrophilic properties study are presented in Figure 7. For the PMA that was not subjected to laser processing, the average contact angle is 61.6°. For the material with pattern 2 (spots with rare craters on the surface), the average angle is 61.2°, which has no statistically significant difference from the untreated polymer. Thus, laser processing with the application of spots did not affect the change in the hydrophilic properties of the surface of the material with pattern 2. However, the material with pattern 1 is characterized by a significant decrease in the contact angle. Creating grooves on the polymer surface using laser radiation significantly improved the hydrophilic properties of the material, and the average contact angle was 40.8°. So, a decrease in the contact angle indicates an increase in the hydrophilicity of the surface after treatment, which may contribute to improved contact interaction between human cells and the surface of the material [51]. For example, in [44], Nd:YAG laser treatment of polymer films contributed to a significant increase in surface wettability and improved cell attachment and growth compared to the unmodified surface of the films. Thus, the high hydrophilicity of PMA with pattern 1 compared to untreated PMA and PMA with pattern 2 may contribute to improving cell biocompatibility and adhesion upon contact with the surface of the material with grooves in our study.

### 3.2. Biocompatibility

#### 3.2.1. Cell Survival Rates Using MTT Assay and Fluorescence Microscopy

Microrelief on the surface of polymeric materials obtained using laser radiation allows us to evaluate how the formed structures influence the bioactive properties of the products. According to [52], changing the surface relief of polymers significantly improves the biocompatibility of the product, making methacrylate resins preferable for use in orthodontics.

**Figure 8:**
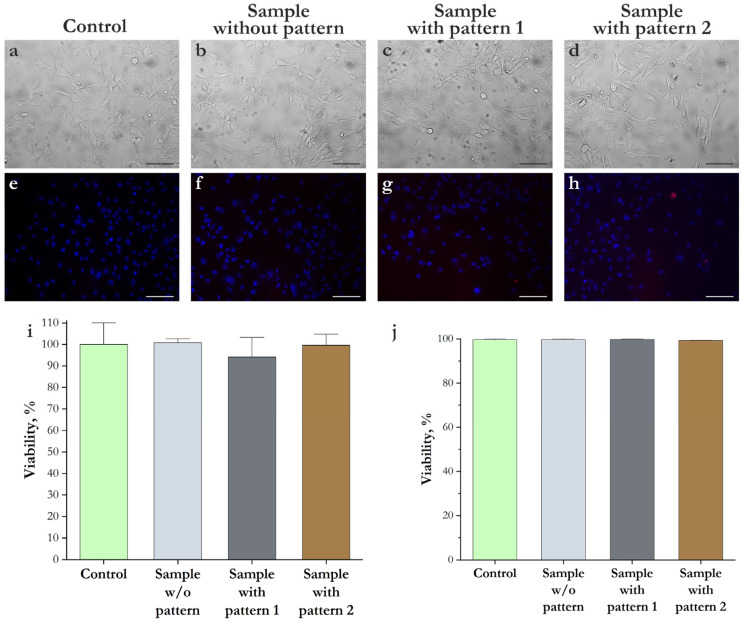
Evaluation of material cytotoxicity against human spleen fibroblasts in vitro after 72 h of culture. Micrographs of HSF cells after 72 h experiment without experimental samples (**a**,**c**), in the presence of a polymer without pattern (**b**,**f**), a polymer with pattern 1 (**c**,**g**), and a polymer with pattern 2 (**d**,**h**). Images were acquired using bright-field microscopy (**a**–**d**) and fluorescence microscopy, specifically capturing signals from Hoechst (blue), and PI (red) channels (**e**–**h**). The scale bar: 100 μm. Cell viability indices are presented using the MTT assay (**i**) and fluorescence microscopy (**j**). Data are presented as mean values ± SE (*n* = 5). Control OD_555_ was taken as 100%.

Using the MTT assay and fluorescence microscopy, we analyzed the cytotoxicity on human spleen fibroblast cell cultures. The cell morphology and viability after 72 h of cultivation with the polymethacrylates are shown in Figure 8. The results of the biological experiments revealed that laser surface modification has no adverse effect on cell survival: the proportion of viable cells in the culture is high (>90%) for both laser-untreated materials and samples with a modified surface, and no statistically significant differences were observed between the groups under investigation.

The MTT assay is known to assess mitochondrial activity, which is characteristic of viable cells. A slight decrease in cellular metabolic activity was observed for the sample with pattern 1; however, this effect did not have a statistically significant impact on the final survival rates (Figure 8i). Fluorescence microscopy data also confirm the high biocompatibility of the studied materials and the absence of a prolonged cytotoxic effect (Figure 8j).

**Figure 9:**
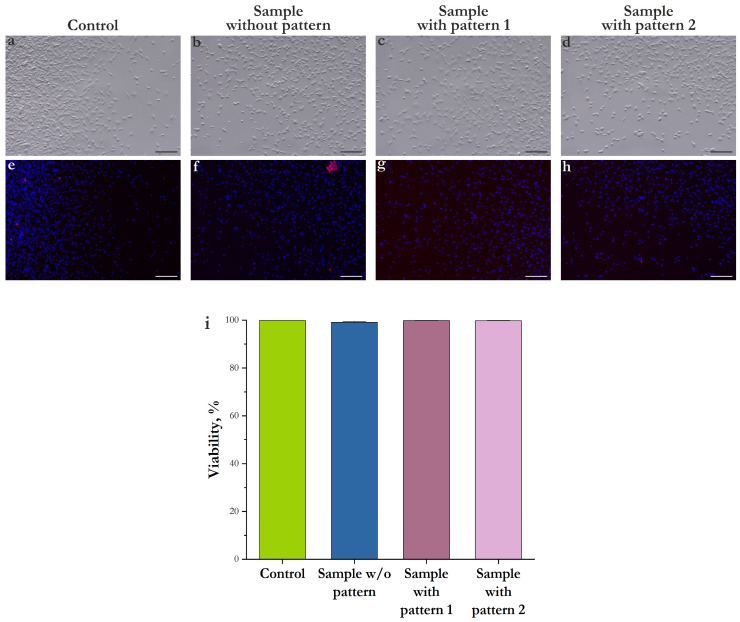
Evaluation of material cytotoxicity against epithelial cells of the human oral mucosa in vitro after 72 h of culture. Micrographs of NCU-F8 cells after 72 h experiment without experimental samples (**a**,**c**), in the presence of untreated PMA (**b**,**f**), PMA with pattern 1 (**c**,**g**), and PMA with pattern 2 (**d**,**h**). Images were acquired using bright-field microscopy (**a**–**d**) and fluorescence microscopy, specifically capturing signals from Hoechst (blue), and PI (red) channels (**e**–**h**). The scale bar: 100 μm. Cell viability indices are presented using fluorescence microscopy (**i**). Data are presented as mean values ± SE (n = 5).

An additional experiment was the assessment of the viability of the fibroblast-like cell line NCU-F8, isolated from human oral mucosal epithelium, using fluorescence microscopy. Under similar conditions (see Section 2.2. Biological tests), the cells were cultured for 72 h in the presence of experimental samples. Exactly the same as in experiments with HSF, the PMAs were placed in close proximity to the cells (without direct physical contact). Results obtained using fluorescence microscopy demonstrate high viability on the studied materials: untreated PMA and PMA with patterns 1 and 2 (Figure 9).

Results for HSF and NCU demonstrate viability of greater than 90% (Figure 9i). Materials, placed near biological objects during cell culture cultivation, are nontoxic to both cell lines. In further experiments, the HSF cell line was selected to evaluate the contact interaction between cells and experimental samples.

#### 3.2.2. Proliferative Activity Assessment Using SEM

Scanning electron microscopy in the backscattered electron mode using lanthanide staining was used to analyze the morphology of human spleen fibroblasts, their differentiation, and the nature of their adhesion to the studied materials. The duration of cell cultures on the studied samples was 24 and 96 h (1 and 4 days). The high contrast of the obtained SEM images characterizes the accumulation of lanthanides in the structures of living cells and allows for the visualization of nucleoli, cell membrane, cytoskeleton, and areas of local accumulation of phosphate anions, which characterize intercellular communication [53]. Thus, lanthanide staining makes it possible to observe the ultrastructure of cells in their ametabolite state and track the features of intercellular interactions [54].

**Figure 10:**
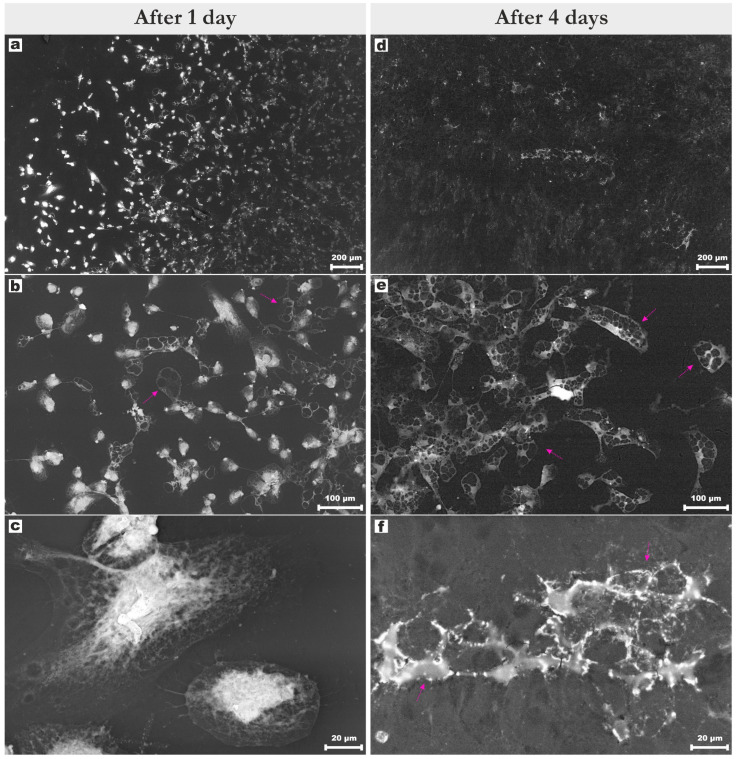
SEM images of the surfaces of the initial polymethacrylate samples (without laser exposure) after experiments with the HSF cell line after 1 day (**a**–**c**) and 4 days (**d**–**f**). Pink arrows indicate cellular debris and blebs.

For polymethacrylate materials whose surfaces were not subjected to laser texturing, after 24 h, the spread of the cellular “front” from the fibroblast adhesive center was observed: the migratory activity of the first generation was replaced by the proliferation of a new generation on the surface of the studied sample (Figure 10a). For the material without texturing, a significant number of areas with metabolically active cells were observed (Figure 10c); however, zones with apoptotic vesicles were visible on the membrane of some fibroblasts, demonstrating morphological signs of cell death. As biological objects migrated to the edge of the material, “blebbing” of the culture—programmed cell death—was observed (Figure 10b), indicating the onset of a contact toxic effect from the sample and the loss of the ability of biological structures to maintain proliferative activity. After four days (96 h), the HSF culture was in a metabolically inhibited state, with pronounced signs of apoptosis (Figure 10d–f). This state of the cells indicates the cytotoxic effect of the polymer material after 96 h.

**Figure 11:**
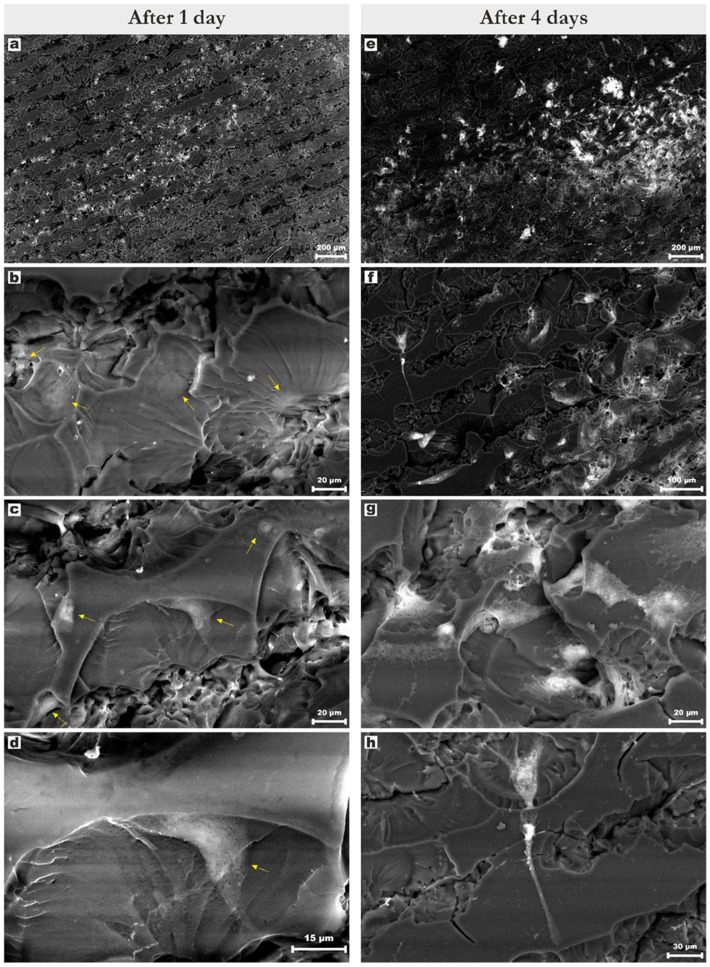
SEM images of the surfaces of polymethacrylate samples with laser pattern 1 after experiments with the HSF cell line after 1 day (**a**–**d**) and after 4 days (**e**–**h**). Yellow arrows indicate HSF cells.

Polymethacrylate materials with surface geometry 1 exhibit high cellular metabolic activity, characterized by a bright contrast in scanning electron microscopy images for both experiments (24 and 96 h) (Figure 11). After 24 h, fibroblasts exhibit their typical spread morphology and demonstrate adhesion along the grooves, establishing strong contact with the irregularities (observed as “shells” in the images) within the grooves. Cells on the sample with pattern 1 do not exhibit a high rate of spread, unlike the material without texture; however, no signs of apoptotic death are observed on the surface of this sample, and the cells are in the adaptation phase. After 4 days, the number of fibroblasts has increased significantly due to their proliferative activity. The cells are spread along the laser-cut depressions, and most have a spindle-shaped form. A pattern of pronounced intercellular contacts is observed, and the biological structures retain their viability. The microrelief of the sample with geometry 1 had a positive effect on the adhesive capacity of biological objects and contributed to a contact reduction in the cytotoxicity of the material.

**Figure 12:**
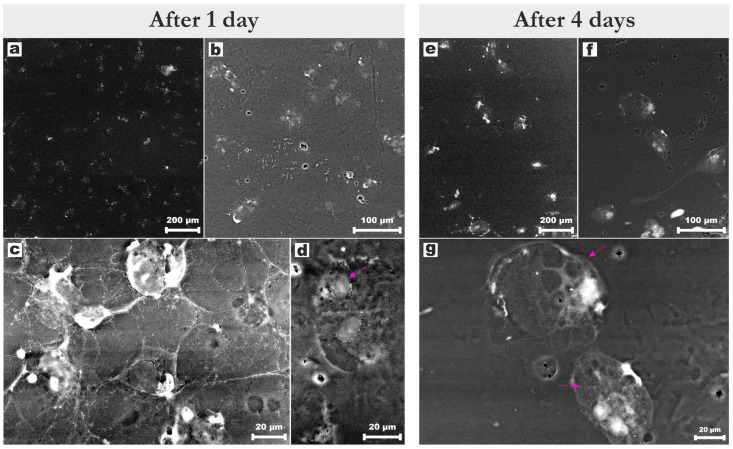
SEM images of the surfaces of polymethacrylate samples with laser pattern 2 after experiments with the HSF cell line after 1 day (**a**–**d**) and 4 days (**e**–**g**). Pink arrows indicate metabolically inhibited cells and blebs.

On polymethacrylate samples with pattern 2, areas of viable cells were observed after 24 h; however, no active colonisation of the material’s surface was noted (Figure 12a–d). After 96 h, cellular debris with clear signs of apoptosis was observed on the surface of the polymethacrylates with the second pattern (Figure 12e–g). Just like the materials without laser texturing, the samples with pattern 2 proved to be toxic to the HSF cell line.

Consequently, the sample with pattern 1 is the most biocompatible with HSF cells under chronic experimental conditions. However, the assessment of PMA hydrophilicity by measuring contact angles offers a mechanistic insight into the improved adhesion of human fibroblasts for PMA with surface geometry 1. The sample with grooves (pattern 1) with an average contact angle of 40.9° (see Section 3.1) exhibited a highly wettable surface, which explains the viability of cells within the laser-induced cavities after 96 h of cultivation (Figure 11e–h). In turn, for PMA with pattern 2, the contact angle was 61.2°, while for untreated PMA, it was 61.6°, which can be classified as moderately hydrophilic surfaces. Laser treatment of PMA, which formed spots with rare craters, did not impart more pronounced hydrophilic properties to the material surface and, upon contact with human fibroblasts, proved toxic to them after just 24 h of culturing (Figure 12c,d). A similar situation was observed for PMA without a pattern. The surface with a water contact angle of 61.6° was moderately hydrophilic and exhibited cytotoxicity, as evidenced by apoptotic signs in cellular structures after 24 h of culturing (Figure 10e). A surface with more pronounced hydrophilic properties (for example, after laser treatment) does improve the attachment of cell cultures, which is consistent with the literature data [43,55,56]. For example, in [44], using laser treatment, the authors reduced the contact angle from 70° to 40°, increasing surface wettability of the experimental samples and thus promoting stronger attachment of fibroblast cells upon contact with materials.

### 3.3. Influence of Materials on Bacterial Growth

**Figure 13:**
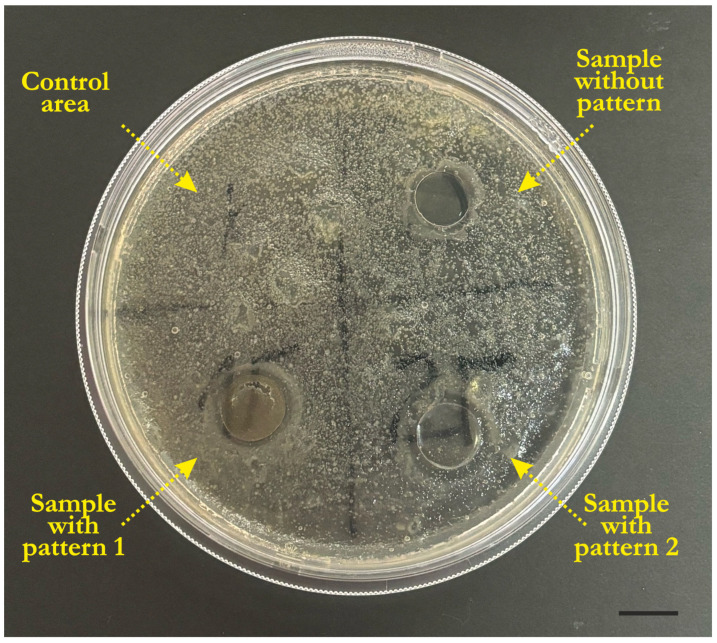
Experimental photograph of the studied samples after the disk diffusion method. Scale bar: 1 cm.

**Figure 14:**
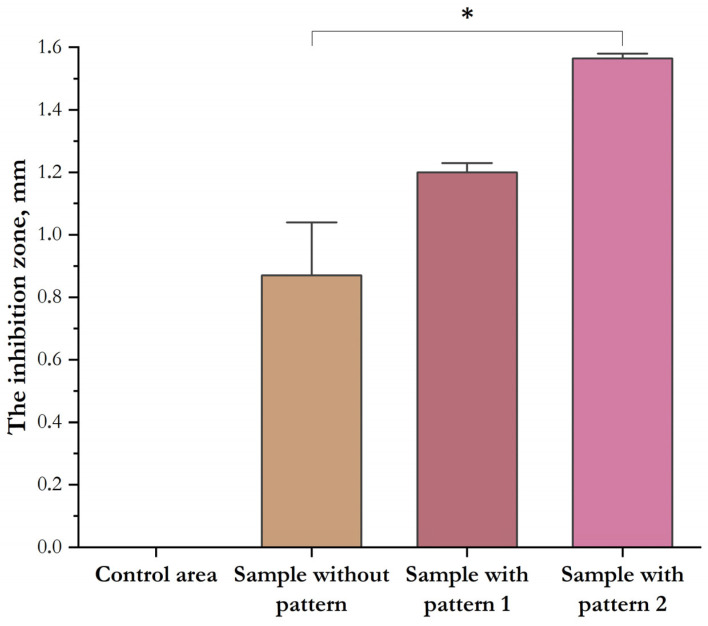
Inhibition zone values against *E. coli* for PMA without pattern and PMA with patterns 1 and 2. Data are presented as mean values ± SE (*n* = 2), *—*p* < 0.001.

The antibacterial activity of the polymethacrylate samples was studied against Gram-negative bacteria using disk diffusion and biofilm formation tests. Figure 13 shows zones of growth inhibition of *E. coli* around the experimental samples after the disc diffusion method. Agar diffusion revealed small inhibition zone values around the experimental materials: 0.9 mm for the sample without geometry, 1.2 mm for the sample with geometry 1, and 1.6 mm for the sample with geometry 2 (Figure 14). Notably, after 24 h, a growth-inhibitory zone appeared only for the polymethacrylate with pattern 1; however, a sample with this surface structure promotes uneven diffusion around it. We also observe a larger inhibition zone for PMA with pattern 2 compared to the untreated sample and PMA with pattern 1. Nevertheless, this method does not allow for a reliable analysis of the antibacterial properties of the polymethacrylates and is limited by the low diffusion capacity of the samples into the agar medium. Subsequently, a method such as the biofilm formation test was used to more accurately assess the impact of materials on *E. coli*.

**Figure 15:**
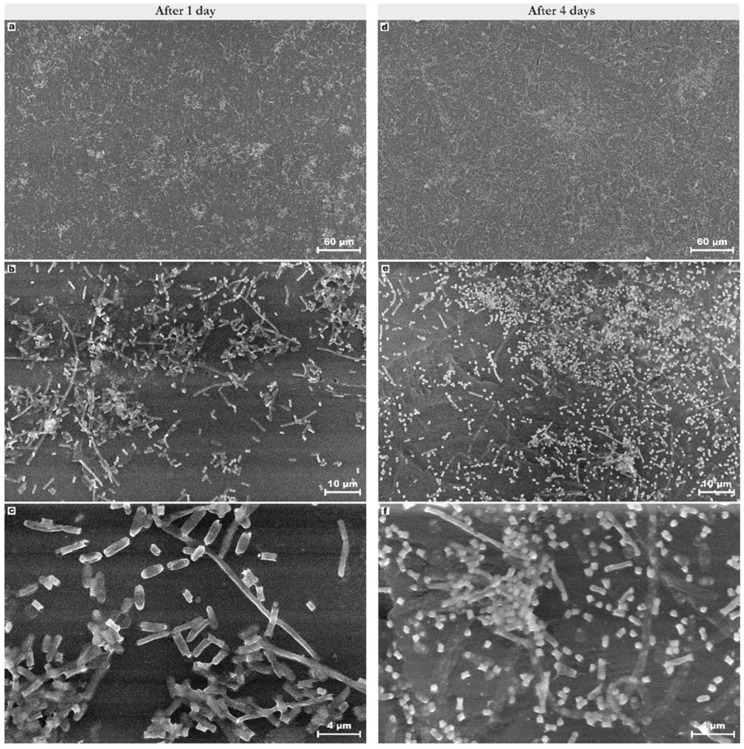
SEM images of formed *E. coli* biofilms on a control sample without a modified surface after 1 day (**a**–**c**) and 4 days (**d**–**f**) at different magnifications.

**Figure 16:**
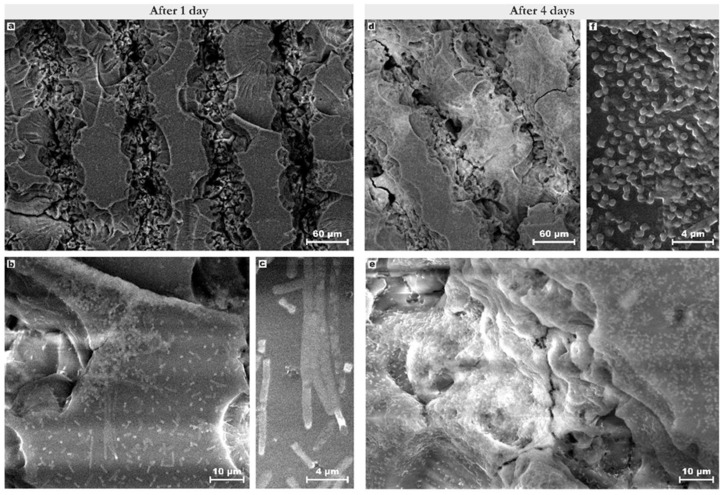
SEM images of formed *E. coli* biofilms on a sample with pattern 1 after 1 day (**a**–**c**) and 4 days (**d**–**f**) at different magnifications.

**Figure 17:**
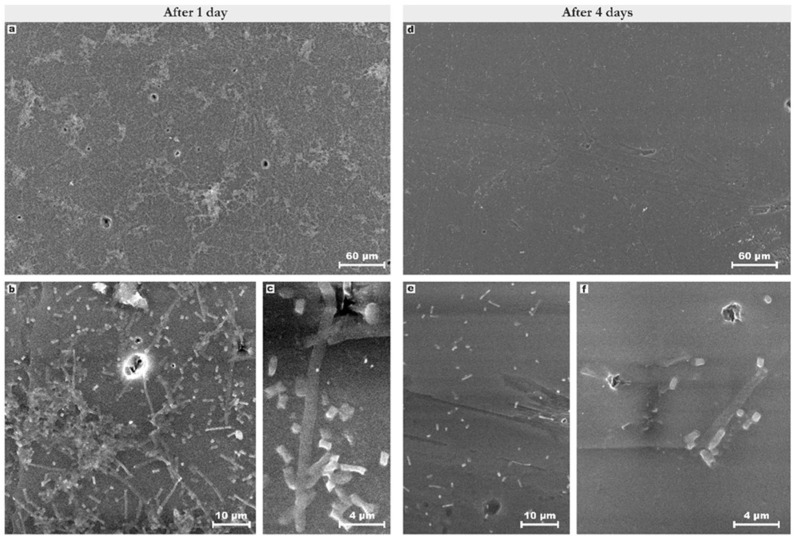
SEM images of formed *E. coli* biofilms on a sample with pattern 2 after 1 day (**a**–**c**) and 4 days (**d**–**f**) at different magnifications.

A study of bacterial biofilm formation using scanning electron microscopy clearly demonstrates the colonization of polymethacrylate surfaces by microorganisms. It is worth noting that, due to the flagella and pili in their structure, *E. coli* bacterial cells have a high capacity to form biofilms on surface structures [57]. However, the results of the experiments revealed differences in the nature of surface overgrowth of the studied samples and the intensity of bacterial growth on them (Figure 15, Figure 16 and Figure 17).

SEM analysis revealed that the unmodified polymer surface was colonized by *E. coli* bacteria at high densities (Figure 15). After just 24 h, the cells demonstrated strong attachment to the surface and secreted extracellular polymeric substances that facilitated the formation of bacterial microcolonies (Figure 15a–c). After 4 days, the polymethacrylate surface was coated with *E. coli* islets, which formed a mature biofilm with a three-dimensional structure.

The adhesion of *E. coli* on the surface of a sample with pattern 1 is shown in Figure 16.

After 24 h of incubation, an accumulation of bacteria is observed both on the microrelief and on the flat part of the surface, and after 96 h, bacterial biofilms have formed (including on the laser-created indentations). This result is explained by the fact that the surface with irregularities created on it (e.g., grooves, pillar structures), the size of which is larger than the size of the bacterial cell itself, increases the contact area between microbes and the surface of the material and promotes the attachment of microorganisms [58,59]. For a more pronounced antibacterial effect, it is preferable to create a nanometer-scale pattern [58].

For material with geometry 2, a different situation is observed compared with the previous samples (Figure 17). After 24 h, active bacterial colonisation of the surface takes place (Figure 17a–c). However, in Figure 17a, it can be seen that the microorganisms form islands of microcolonies away from the round-shaped depressions created on the sample surface by laser irradiation. This behavior can be explained by the presence of unstable conditions for microbial attachment: the size of the craters, which is smaller than that of the bacteria, reduces the microorganisms’ ability to attach firmly [60,61]. After 96 h, scanning electron microscopy revealed a significant reduction in the bacterial population on the surface of the material with surface geometry 2. It can be assumed that this surface topography exhibits an anti-fouling effect and reduces the adhesive properties of the cells. Nevertheless, microtopographical patterns with proven antifouling performance have been reported in the literature. For instance, the sharkskin topography, whose structure size exceeds the size of a bacterial cell, exhibits an anti-fouling effect. It can be explained by the following mechanism: the structural features of sharkskin relief can prevent the interaction of neighboring cells and the subsequent formation of bacterial islets and/or biofilms [62]. However, fabrication typically relies on photolithography and dry etching, which differ fundamentally from the picosecond laser ablation employed in our study. The present study did not aim to replicate or directly compare with this topography, but obtaining such structures using laser ablation represents a promising direction for future research.

**Figure 18:**
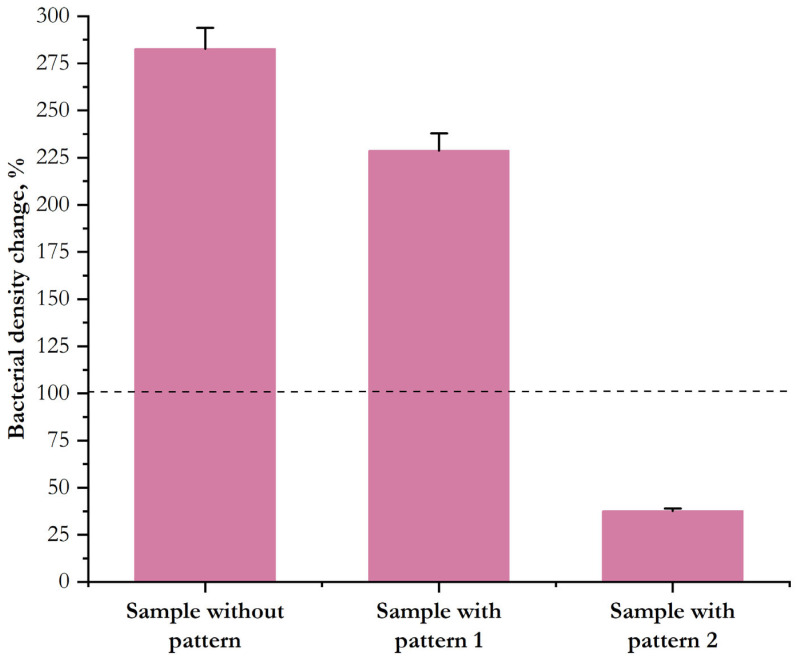
The percentage change in bacterial culture density between 1 and 4 days of cultivation for the surfaces of the control polymethacrylate (without pattern) and polymethacrylates with patterns 1 or 2. Bacterial density on each sample after 1 day was accepted as 100% (dashed line).

SEM images were used to automatically count bacterial cells and *E. coli* growth density on the surface of materials using ImageJ 1.54p software. A macro was developed to identify microbial cell regions and islets, determining their number, area, and length. For the analysis, images of the control polymer at a magnification of 10 μm, the polymer with pattern 2 at a magnification of 10 μm, and the polymer with pattern 1 at a magnification of 4 μm were used. Figure 18 shows the percentage change in bacterial culture density between 1 and 4 days of cultivation for an untreated polymethacrylate surface and polymethacrylate surfaces with patterns 1 and 2. It was found that after 4 days, compared to 1 day, the bacterial count increased by 2.8 times for the polymethacrylate without a pattern, by 2.3 times for the polymethacrylate with pattern 1, and decreased by 2.6 times for the polymethacrylate with pattern 2. These data suggest that the antibacterial properties of the material with geometry 1 are slightly lower than those of the original polymer, while the material with geometry 2 demonstrates a significant reduction in the density of *E. coli*. Direct contact of bacteria with polymethacrylate surfaces is what causes differences in microbial adhesion and biofilm formation. —a decrease in cell density on the surfaces of polymers with patterns 1 and 2 compared to the control polymer.

A similar situation was observed for HSF. SEM analysis revealed that the surfaces of the control sample (without laser treatment) and the sample with pattern 2 were toxic to fibroblasts when in contact with them for more than 24 h (see Section 3.2.2). However, cell viability indicators determined using the MTT assay and fluorescence microscopy showed that the experimental samples incubated in the presence of cells do not release toxic components and are safe for the surroundings (see Section 3.2.1).

## 4. Conclusions

In this study, the surface of methacrylate-based polymers was modified using laser radiation. Two geometric patterns were obtained—grooves and spots in the near-surface layer, with occasional rounded depressions extending to the surface. Using optical microscopy and SEM, it was established that the control polymer had a smooth surface, while the material with pattern 1 had furrows 75 µm wide, spaced 45 µm apart, and the material with pattern 2 had spots 30 µm in diameter, arranged along the material at 80 µm intervals. Evaluation of the surface hydrophilicity revealed that PMA with pattern 1 exhibited increased wettability following laser-induced surface grooving, with a water contact angle of 40.8°. Using the MTT assay and fluorescence microscopy, it was found that all materials are cytocompatible, and the viability of cell cultures on their surfaces is high. In turn, scanning electron microscopy allowed us to assess the morphology and proliferative activity of fibroblasts on the experimental samples. It was established that laser etching of a groove-like pattern onto the surface of polymethacrylate promotes improved cell attachment to the material and a reduction in contact cytostatic activity compared to untreated material and the sample with geometry 2. At the same time, the sample with geometry 2 possesses “anti-fouling” properties, preventing the formation of a bacterial biofilm, in contrast to the control sample and the material with geometry 1. Thus, laser surface treatment of PMA materials enables a notable improvement in biocompatibility and ensures enhanced integration with biological structures. Modification using photonic technologies allows the surface structure of polymers to be altered in a controlled manner to achieve the desired cellular behavior. The use of materials with laser-induced surface micro-relief demonstrates high potential for application in dentistry, ensuring improved adhesion to cells and tissues of the oral cavity. The present study has several limitations. First, the 1 kHz repetition rate used in this laboratory setup is not sufficient for industrial-scale processing of complex 3D dental appliances. Translation to production would require higher-repetition-rate systems, optimized scanning strategies, and focus-tracking approaches to compensate for curved transparent surfaces. These concerns should be addressed in future works, via measures including direct quantitative comparison with other advanced surface modification techniques (e.g., femtosecond laser or nano-patterning), to benefit both research and industrial purposes.

## Data Availability

The raw data supporting the conclusions of this article will be made available by the authors on request.

## Abbreviations

The following abbreviations are used in this manuscript:

AFM	Atomic force microscopy
DMSO	Dimethyl sulfoxide
FBS	Fetal bovine serum
HSF	Human spleen fibroblasts
LB	Lysogeny broth
MHA	Mueller-Hinton agar
MPC	2-methacryloyloxyethyl phosphorylcholine
MTT	3-(4,5-Dimethylthiazol-2-yl)-2,5-diphenyltetrazolium bromide
PBS	Phosphate-buffered saline
PC	Polycarbonate
PHAs	Polyhydroxyalkanoates
PI	Propidium iodide
PMA	Polymethacrylate
PMMA	Poly(methyl methacrylate)
SEM	Scanning electron microscopy
